# Professional development: Training in ethics

**DOI:** 10.1192/j.eurpsy.2021.1583

**Published:** 2021-08-13

**Authors:** O. Shchedrinskaya, M. Bebtschuk, E. Snarskaya

**Affiliations:** Science, Moscow State Budgetary Health Care Institution “Scientific and Practical Center for Mental Health of Children and Adolescents named after G.E. Sukhareva of Moscow Health Department, Moscow, Russia, Moscow, Russian Federation

**Keywords:** ethical dilemmas, training, application of ethics, ethics

## Abstract

**Introduction:**

Some of the most vulnerable psychiatry patients are children under 18, as they can’t protect themselves and their rights from unethical behaviour of mental health professionals. There is a gap between theoretical knowledge and application of ethics at the workplace. Continuous education in ethics is necessary to address this gap.

**Objectives:**

The objective of the study was to compare various forms of education in ethics and develop training for mental health practitioners.

**Methods:**

The study had 2 groups (356 participants, aged 23 to 67, average age – 41.3) – staff of the main and oldest children’s mental health clinic in Moscow, Russia. The control group (124 participants) of mental health professionals received written materials on ethics (such as ethical codes and ethical decision-making protocols). The test group (232 people) participated in a several trainings on ethics. The trainings included 3 parts – discussing the code of ethics, creating examples of potential ethical challenges and role-plays. Participants reported that the topics on the quality of care, common ethical dilemmas and relationships between the practitioners, young patients and legal guardians, were the most helpful for them.

**Results:**

The survey was conducted to evaluate the results. 70.8% of staff members that participated in training shared that they feel confident about applying the Code and the decision-making protocols in unclear cases. Only 32.6% from the control group reported the same level of confidence.

**Conclusions:**

Hands-on training in ethics for continuing education has shown to be more beneficial, as compared to theoretical instructions

